# Ionothermal synthesis of activated carbon from waste PET bottles as anode materials for lithium-ion batteries†

**DOI:** 10.1039/d2ra06786b

**Published:** 2022-12-02

**Authors:** Cyril O. Ehi-Eromosele, Chizoom N. Onwucha, Samuel O. Ajayi, Georgian Melinte, Anna-Lena Hansen, Sylvio Indris, Helmut Ehrenberg

**Affiliations:** Institute for Applied Materials (IAM), Karlsruhe Institute of Technology (KIT) 76344 Eggenstein-Leopoldshafen Germany cyril.ehi-eromosele@covenantuniversity.edu.ng; Department of Chemistry, Covenant University PMB 1023 Ota Nigeria; Helmholtz Institute Ulm (HIU) Electrochemical Energy Storage Helmholtzstrasse 11 89081 Ulm Germany; Institute of Nanotechnology (INT), Karlsruhe Institute of Technology (KIT) Hermann-von-Helmholtz-Platz 1 76344 Eggenstein-Leopoldshafen Germany

## Abstract

Waste polyethylene terephthalate (PET) bottles have become a significant post-consumer plastic waste with attendant environmental problems. Hence, ionothermal synthesis has been used to prepare activated carbon (AC) anode materials from waste PET for both high performance and sustainable lithium-ion batteries (LIB). Particularly, using choline chloride deep eutectic salts (CU-DES) does not require post-synthesis washing and thereby reduces the complexity of the process and produces materials with unique low-surface area, higher levels of graphitization/ordering, and high nitrogen doping in the obtained ACs. The results show that the AC produced using CU-DES (PET-CU-A-ITP2) gave good electrochemical performance. Even though the material possesses a low surface area (∼23 m^2^ g^−1^), it displays a gravimetric capacity (GC) of ∼460 mA h g^−1^ and a coulombic efficiency (CE) of ∼53% in the 1^st^ cycle and very good cycling performance with a capacity retention of 98% from the 2^nd^ to the 100th cycle. The superior electrochemical performance of the PET-CU-A-ITP2 anode was found to be due to its better graphitization/ordering and dense structure which results in higher capacity, formation of less solid electrolyte interphase, and higher CE. These results show that dense carbons can be exploited as high-performance anodes in LIBs. Also, this research presents both a pathway for waste PET management and a waste-energy approach that could offer cheaper and greener LIBs to meet the sustainable development goals.

## Abstract 2

PET-derived activated carbons prepared by a facile method are promising anodes for sustainable lithium-ion batteries and ensure PET circularity.

## Graphical abstract

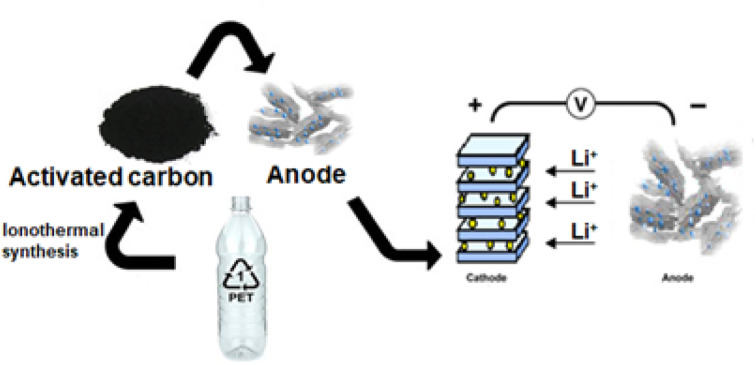

## Introduction

The most effective energy storage system (ESS) for renewable energy is a lithium-ion battery (LIB) owing to its unparalleled high energy density, high power density, better portability, and non-memory effect when comparing it with other energy storage devices.^1,2^ As a result, it is the most suitable source of power for both portable devices such as smartphones, calculators, laptops, digital cameras, *etc.* and larger energy storage systems such as electrical grids and electric vehicles. LIBs are electrochemical rechargeable devices that involve the exchange or shuttling of lithium ions between the positive electrode (cathode) material and negative electrode (anode) material while the electronic current flows across the external load.^3–8^

One of the most important components of LIBs and other ESS are the electrodes. Suitable electrode materials with novel structure are being exploited to improve their performance. This includes the preparation of electrode materials coupled with the development of synthesis methods that can tailor the required textural properties of the materials; and also provide greener and cheaper synthetic routes.^9–11^ Porous carbon-based materials find extensive applications in ESS because of their abundance, chemical and thermal stability, excellent conductivity, low weight, and tunable pore structure. Specifically, activated carbons (ACs) are very attractive due to their large specific surface area (SSA), large pore-volume, low-cost, and well-established methods for their preparation.^12^ Porous carbonaceous materials are mostly obtained from fossil-based carbon sources making them costly and environmentally unsustainable.^12^ Therefore, it is imperative to exploit and develop renewable derivatives and/or products to prepare porous ACs through economically viable and environmentally friendly approaches (*i.e.*, through waste-to-value added product concept). Biomass materials, as cheap and sustainable precursors for the preparation of porous ACs, have been exploited as electrode materials in LIBs to attain a circular economy.^12–14^

Similarly, other abundant waste sources such as waste plastic can serve as cheap and sustainable starting-materials for the synthesis of porous ACs.^15–18^ Of particular interest in this regard is polyethylene terephthalate (PET) since it is a ubiquitous, non-biodegradable post-consumer polymer waste associated with severe environmental problems worldwide. In addition, the high amounts of carbon coupled with the small amounts of impurities in PET make it an attractive precursor for the preparation of ACs.^16^ Thus, converting PET wastes into valuable chemical products such as AC material has become an attractive way to not only manage PET wastes sustainably but most importantly, to achieve a circular economy. This suggests that PET bottles could serve as a raw material for the synthesis of porous AC which could find application in separation,^19^ catalysis,^20^ hydrogen storage,^21^ and as electrode materials in supercapacitor.^22^

Conventionally, there are two routes used for the preparation of porous ACs from PET wastes namely, the physical and chemical activation methods.^16,18^ The former one involves exposing the carbon precursor (PET wastes) to oxidizing gases such as water steam or CO_2_ at high temperature (usually between 600–1200 °C), while for the latter method, the carbon precursor is impregnated with an activating agent (KOH, NaOH or ZnCl_2_, *etc.*) before thermal treatment under inert conditions. Usually, before the application of either the physical or chemical activation methods, the PET waste is first converted into a carbon-rich matter through thermal treatment or pyrolysis. Though the physical activation method is environment-friendly, it leads to reduced particle and micro-domain sizes in the ACs which may be deleterious to the performance of the material. Moreover, the activation temperature is very high which makes the process energy and cost intensive. Comparatively, chemical activation gives lower temperatures of activation, shorter activation time, higher yields and higher specific surface area (SSA) over physical activation. Nevertheless, it still results in low yields of carbon and involves tedious washing processes (using large amounts of acid and water) to prevent corrosion. Also, the usage of activation reagents is excessive thereby limiting its commercial development and application.

Therefore, it is desirable to develop novel routes that are green, scalable and inexpensive for the efficient synthesis of porous ACs from abundant waste PET bottles. The ionothermal synthesis (IS) process combines simplicity, scalability, high yields and the possibility to tune the porosity of the AC materials. In IS, ionic liquids or deep eutectic salts (DES) are used as solvents or additives to improve the kinetics of carbonization/activation and to further increase the SSA of AC materials.^23–25^ This one-pot synthesis to prepare highly porous ACs simply requires aqueous washing of the as-obtained product for removal of the inorganic salts (DES or ionic liquids) after carbonization/activation of the carbon precursor. IS strategy has been used to obtain ultra-high yield porous AC from the pyrolysis of sugarcane bargasse soaked initially in ZnCl_2_/urea/KCl DES.^25^ Choline chloride–urea (CU) DES has been used as a dissolution solvent due to its solvation ability stemming from its rich hydrogen bond acceptors which can break intra- and inter-molecular hydrogen bonds.^26^ Particularly, CU-DES has been used in the valorisation of lignocellulosic biomass for delignification or breaking down its recalcitrant structure.^27^ Furthermore, since CU-DES contain rich nitrogen sources it can help to dope high amounts of nitrogen into the final carbon material framework, thereby improving its conductivity and electrochemical performance. Owing to these findings, CU-DES could be used as a solvent medium to prepare AC from waste PET bottles. It is entirely an organic solvent which can help increase the yield of the final AC material since it can also serve as a carbon source. For this reason, it is hypothesised that the use of CU-DES will not require any post-synthesis washings usually done with inorganic activating salt containing DES since it is expected to completely be degraded after carbonisation. Herein, a green ionothermal pyrolysis synthetic route using CU-DES is proposed to prepare ACs from waste PET bottles. Unlike most reports, no post-synthesis washing of the as-synthesised ACs was undertaken. Therefore, CU-DES can help to reduce the cost, complexity and toxicity of producing AC from waste PET, instead of using conventional activating chemicals, which is one of the motivations for this research. Also, this method produced high nitrogen-doped ACs that were dense and of appreciable yield which were then used as anode materials in LIB.

## Experimental section

### Materials

Post-consumer waste PET bottles were collected, air-dried and shredded to particle sizes of between 4 and 5 mm. The shredded PET pieces were washed again with water and air dried and stored in Ziploc bags and labelled PET-P. Choline chloride (98+%) was obtained from Alfa Aesar, urea from Merck, and other chemicals such as polyvinylidene fluoride (PVDF) and *N*-methyl-2-pyrrolidone (NMP) were obtained from Sigma Aldrich. Coin-type cells, Celgard separators, graphite (TIMCAL C-NERGY SFG 6 L), LP30 selectylite (BASF) electrolyte, aluminium, copper and lithium metal foils were used for electrode coating and coin cell assembling prior to electrochemical measurements.

### Ionothermal synthesis of activated carbons from waste PET bottles

ACs were prepared by ionothermal pyrolysis (ITP) of waste PET bottles following the modified procedures of Zou *et al.* and Pambel *et al.*^23,25^ The first step consists of the preparation of different mixtures of deep eutectic salt (DES). Choline chloride and urea were mixed in a molar ratio of 1 : 2 inside a conical flask. The salt mixture, placed in an oil bath, was heated at 80 °C for 3 h with magnetic stirring at 500 rpm. The DES obtained was a clear homogeneous solution which was stored in a glass bottle and labelled CU-DES. In a typical procedure, 7.0 g of PET-P was mixed with 20 mL of CU-DES at room temperature to obtain the precursor (labelled PET-CU) that was used for both the annealing and pyrolysis carbonisation step. In the annealing step, PET-CU was placed in a muffle furnace and heated to 400 °C at a rate of 2 °C min^−1^ under air and this temperature was maintained for additional 2 h. Shiny black and coarse carbonaceous material was obtained which was ball-milled into fine powders at 500 rpm for 3 h using a FRITSCH ball-mill. The obtained powders were labelled PET-CU-A and used for further analysis. For comparison, the PET-P without impregnation with CU-DES was also annealed following the step above and was labelled PET-P-A. In the pyrolysis step, PET-CU was first stabilised by heating to 380 °C at a rate of 2 °C min^−1^ under air and this temperature was maintained for additional 4 min. After this short annealing step, the obtained material was ground and pyrolysed at 800 °C at a rate of 5 °C min^−1^ under nitrogen flow (0.55 L min^−1^) and this temperature was maintained for additional 1 h to carbonise and activate it. The obtained carbonaceous material was ball-milled into fine powders at 500 rpm for 3 h and it was labelled PET-CU-A-ITP2. For comparison, PET-P, without impregnation with CU-DES, was annealed and pyrolysed following the step above and was labelled PET-P-A-ITP2.

### Characterization

The product yields (%) were determined by obtaining the masses of the precursor (PET-P) and the obtained dried AC materials. The elemental analysis of the ACs were undertaken using a Vario micro cube from elementar with C, H, and N determined with the thermal conductivity detector while the value of S was detected by infrared detector. The SSA of the AC materials were measured by nitrogen adsorption isotherms at low temperatures (77.4 K) on a volumetric adsorption analyser (Micromeritics, Gemini VII version 5.03). Before the analysis, the samples were degassed under vacuum at 423 K. The SSA was obtained from the adsorption isotherms using the Brunauer–Emmett–Teller (BET) method. Thermogravimetric Analysis (TGA) coupled with Differential Scanning Calorimetry (DSC) was used to monitor the carbonisation/activation of PET-P and PET-CU samples using Simultaneous Thermal Analysis with combined FT-IR gas analysis operated under Ar-atmosphere (Netzsch Jupiter 449C) from 35–900 °C using a heating rate of 10 °C min^−1^. The structural characteristics of the ACs were obtained from their XRD patterns using a STOE Stadi P laboratory diffractometer equipped with a Cu source and with transmission geometry. Also, total scattering data of selected samples were collected on a STOE Stadi P diffractometer (Ag K_α1_ radiation, *λ* = 0.559 Å), equipped with two MYTHEN flat plate detectors (*Q*_max_ = 18 Å^−1^, *Q*_damp_ = 0.011 Å^−1^). A NIST660b LaB_6_ standard and an empty capillary were measured under the same conditions to account for instrument contribution and to subtract the contribution from the glass capillaries, respectively. All samples were measured in 1.0 glass capillaries (Hilgenberg #10 glass). The calculation of the corresponding pair distribution functions was performed using the program pdfgetX3.^28^ In addition, a HORIBA LabRam Evolution HR (Laser wavelengths: HeNe-Laser (633 nm, 17 mW) Raman microscope was used to determine the degree of order of the carbons in these materials. The morphologies and elemental mapping of C, N, and O in the ACs were obtained by SEM-EDX Merlin microscope (Zeiss GmbH) and TEM scanning mode using a FEI Titan 80–300 microscope.

### Electrochemical measurements

The anode was prepared from a mixture of active materials (AC materials), carbon black, and PVDF in NMP in a weight ratio of 8 : 1 : 1, respectively after stirring the mixture for 4 h. The resulting slurry was coated on a copper foil with a wet thickness of 150 μm. The obtained material was dried in the vacuum oven at 80 °C overnight. This coated electrode film was cut into a disc (12 mm in diameter) for further tests. The electrochemical properties of the discs were tested using 2032 coin-type cells which were assembled in a glovebox (MBraun) flushed with argon gas. To assemble half cells, the as-prepared anode was used as the working electrode and lithium metal foil as the counter electrode which were separated by two Celgard 2325 (16 mm) separators, while BASF LP30 selectylite (1 M LiPF_6_ in a 1 : 1 volume ratio of ethylene carbonate and dimethyl carbonate) was used as the electrolyte.

The galvanostatic charge–discharge tests were performed in the voltage range of 0.01 and 2.8 V (*vs.* Li/Li^+^). The rate capability was measured by varying the cycling current density between 0.1–2.0 A g^−1^ for about 50 cycles. The cycling performance was measured by cycling the cells using a current density of 0.1 A g^−1^ for 100 cycles. The cyclic voltammetry measurements were performed using a scan rate of 0.1 mV s^−1^ in the voltage window of 0.1–2.8 V (*vs.* Li/Li^+^). A VMP3 multichannel potentiostat (Biologic, France) operating at 25 °C was used for all electrochemical tests.

## Results

### Optimisation of the ionothermal synthesis processes

The well-established method of obtaining AC from biomass and PET wastes is chemical activation which is usually undertaken in a single-step with pyrolysis to make the process less cumbersome.^15–18,29^ The synthesis of AC from waste PET bottles was done using the ionothermal pyrolysis (ITP) synthesis route. Deep eutectic salts (DES) have been used as eco-friendly solvents and porogens for the synthesis of AC from biomass.^23,25^ CU-DES has been the most widely used DES and the first to be studied as both choline and urea are naturally derived, biodegradable, very cheap and abundant.^30^ Choline chloride and urea forms a DES when mixed in a molar ratio of 1 : 2 and at low temperature (*i.e.* 80 °C).^31^

**Fig. 1:**
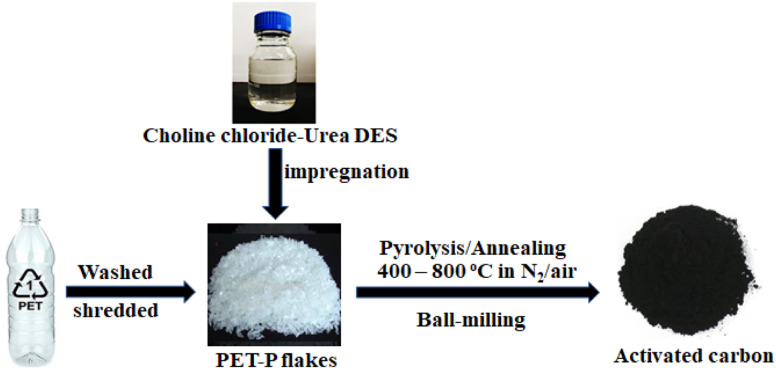
Schematic of the ionothermal pyrolysis of PET waste to obtain activated carbons.

In the ITP process (as shown in Fig. 1), different operating parameters such as the impregnation with/without CU-DES, carbonisation/pyrolysis temperatures and durations, and pre-annealing/stabilisation temperatures both in air and nitrogen flow were optimised. Typically, different carbonisation/pyrolysis temperatures and duration were used to optimise the prepared AC materials. When PET-P and PET-CU were pyrolysed at 800 °C for 2 h under nitrogen atmosphere, the final products were mostly lost by sublimation while the residue had milkish colour. The same behavior was observed when the same precursors were annealed in a muffle furnace in the presence of air. Amorphous carbon with no clear characteristic graphite peaks in the XRD patterns were obtained when the pyrolysis temperature was reduced to 400 °C; the XRD reflections of the obtained carbons (PET-CU-P and PET-P-P) were very low in intensity and broad. However, when these precursors were annealed in the presence of air at 400 °C for 2 h, charred black products were obtained with XRD showing amorphous carbon with the two characteristic graphite peaks, though with low degree of graphitisation. These samples were named as PET-CU-A and PET-P-A. Notably, there was no smoke during PET-CU annealing (seen in PET-P) which can be traced to the presence of CU-DES. Since the previous pyrolysis/annealing treatments, mostly at 800 °C, resulted in the loss (sublimation) of the products, a stabilisation of the precursor at a lower temperature was introduced. It has been reported that polymeric materials such as PET require heating at low temperatures to transform into a stable structure that could withstand the high temperature pyrolysis treatment to form turbostratic carbons.^32^ Furthermore, Ko *et al.* suggested that the presence of oxygen in the structure of PET helps to withstand decomposition so that it can be carbonised as the oxygen atoms serve as linkages. Herein, PET-P and PET-CU were annealed at 380 °C for 4 min in air before transferring to a tube furnace for pyrolysis up to 800 °C for 1 h in nitrogen gas.^33^ No sublimation was seen with these pre-stabilised samples. These carbonaceous materials were named PET-P-A-ITP2 and PET-CU-A-ITP2, respectively. The results show that PET could either be carbonised or stabilised at low temperatures in the presence of air for extended and short-term durations, respectively to obtain carbonaceous materials (after pyrolysis for the pre-stabilised PET).

## Material characterization

### Thermal analysis

**Fig. 2:**
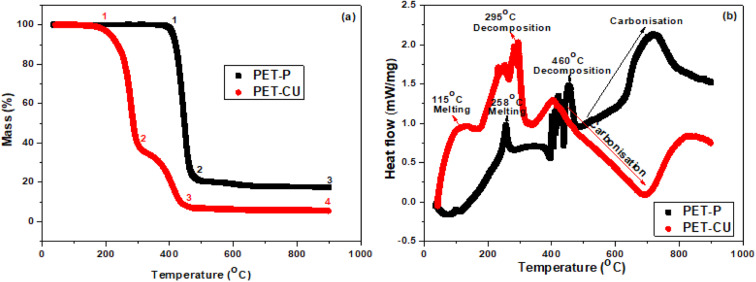
Thermal analysis of PET-P and PET-CU samples. (a) TGA and (b) DSC profiles.

The thermal decomposition processes of PET-P impregnated with and without CU-DES were investigated by TGA and DSC. Fig. 2a shows the TGA profile obtained for the thermal analysis of PET-P and PET-CU between 35 and 900 °C, at a heating rate of 10 °C min under argon gas. The TGA profiles show that both samples had different decomposition stages labelled 1–4. The profile of PET-P shows two different decomposition stages with the first decomposition occurring at a much higher temperature and produced 23 wt% residue (between 1 and 2) while the subsequent carbonisation (between 2 and 3) resulted in 17 wt% carbonaceous materials. The profile of PET-CU shows three different decomposition stages with the first decomposition occurring at a lower temperature to produce 39 wt% residue (between 1 and 2), the second decomposition (between 2 and 3) produced 8 wt% residue which did not further change even after carbonisation (between 3 and 4). The second decomposition which is absent in the profile of PET-P could be due to the decomposition of the CU-DES which contributes to the enhancement of nitrogen doping in the final carbon material as will be shown in the elemental analysis. Also, the reduced initial decomposition temperature of the PET-CU compared to the PET-P indicates the influence of CU-DES in reducing the synthesis temperature for the preparation of AC.

Fig. 2b shows the DSC profiles of the heat flow when PET-P and PET-CU were heated at temperatures between 35 and 900 °C. Three different endothermic peaks corresponding to the degradation regimes in their TGA profiles are observed for both samples. The melting, decomposition, and carbonisation occurred at much lower temperatures in the PET-CU sample compared to the PET-P. It can be seen that CU-DES helped to reduce the decomposition temperature of PET (as shown in the TG) and to reduce the carbonisation temperature, which could increase the yield of the final carbon material. CU-DES like other DES systems has rich hydrogen bond acceptors which can break intra- and inter-molecular hydrogen bonds thereby enhancing its dissolution ability.^26^ Therefore, CU-DES can aid the decomposition of PET which can be seen in the reduced carbonisation temperature of PET-CU compared to PET-P as shown in the DSC result.

### Product yield and elemental analysis

The heat treatment conditions employed during ITP of PET, elemental analysis of obtained carbonaceous materials, and product yield are given in Table 1. The product yields of the CU-DES impregnated PET samples were slightly higher than for the pristine samples. The PET-CU-A and PET-P-A gave product yields of 17% and 15%, respectively. Furthermore, PET-CU-ITP pyrolysed at 400 °C gave a yield of 28% while PET-CU-A-ITP2 that was pre-annealed at 380 °C before pyrolysing at 800 °C gave a yield of 20%. The additional heat treatments resulted in lower yields probably due to structural rearrangement with the loss of volatiles and gases such as CO, CO_2_, NO_2_, CH_4_, *etc.*^34^

**Table 1:** Carbonisation conditions, yield, and elemental analysis of PET-derived carbonaceous materials prepared by ionothermal pyrolysis (ITP) using choline chloride-urea (CU) deep eutectic solvents

Sample	Temperature (^°^C)		Yield (%)	Elemental analysis (weight %)				
	Annealing (A)	Ionothermal pyrolysis (ITP)		C	H	N	S	Residual
PET-P-A	400	—	15	70.10	2.69	3.79	0.00	23.42
PET-CU-A	400	—	17	65.89	2.83	15.72	0.00	15.56
PET-P-ITP	—	400	22	79.26	3.36	0.83	0.06	16.49
PET-CU-ITP	—	400	28	72.37	4.48	15.28	0.09	7.78
PET-P-A-ITP2	380	800	19	95.75	0.00	0.88	0.00	3.37
PET-CU-A-ITP2	380	800	20	89.98	0.44	7.33	0.00	2.25

The elemental analysis generally shows that increasing the temperature in ITP resulted in significant increase in the carbon content and corresponding decrease in the hydrogen content. PET-P-A annealed at 400 °C gave 70.1 wt% C and 2.7 wt% H while PET-P-A-ITP2 that was pre-annealed at 380 °C before pyrolysing at 800 °C gave 95.8 wt% C and no H. These results agree with previous studies on the evolution of C and H contents with carbonisation of lignocellulosic materials.^34,35^ PET-CU samples gave very high amounts of nitrogen in the final carbonaceous products ranging from 7.3–15.7 wt%, which are the highest reported for most nitrogen-doped carbon materials. The high nitrogen content results from urea which is a component of the CU-DES which can be doped into the carbon structure as also reported in the ionothermal synthesis of carbon derived from biomass waste using zinc chloride-urea DES.^25^ There is a decrease in the nitrogen content with temperature. PET-CU-A-ITP2 pre-annealed at 380 °C before pyrolysing at 800 °C displays 7.3 wt% while PET-CU-A annealed at 400 °C gives 15.7 wt%. The residual weight fraction is an indicator of the oxygen content which has been shown to reduce along with hydrogen with higher carbonization temperature.^34–36^ The PET-CU samples give smaller amounts of the residual content compared to the PET-P samples. Since the C and H contents of the PET-P samples show higher carbonization temperatures, the PET-CU samples might require higher heat treatments compared to their PET-P analogues for optimal carbonization. The PET-P-A-ITP2 and PET-CU-A-ITP2 products show the highest amount of carbon, lowest amounts of hydrogen and residual content indicating the highest level of carbonization.

### Structural analysis

#### X-ray diffraction (XRD)

**Fig. 3:**
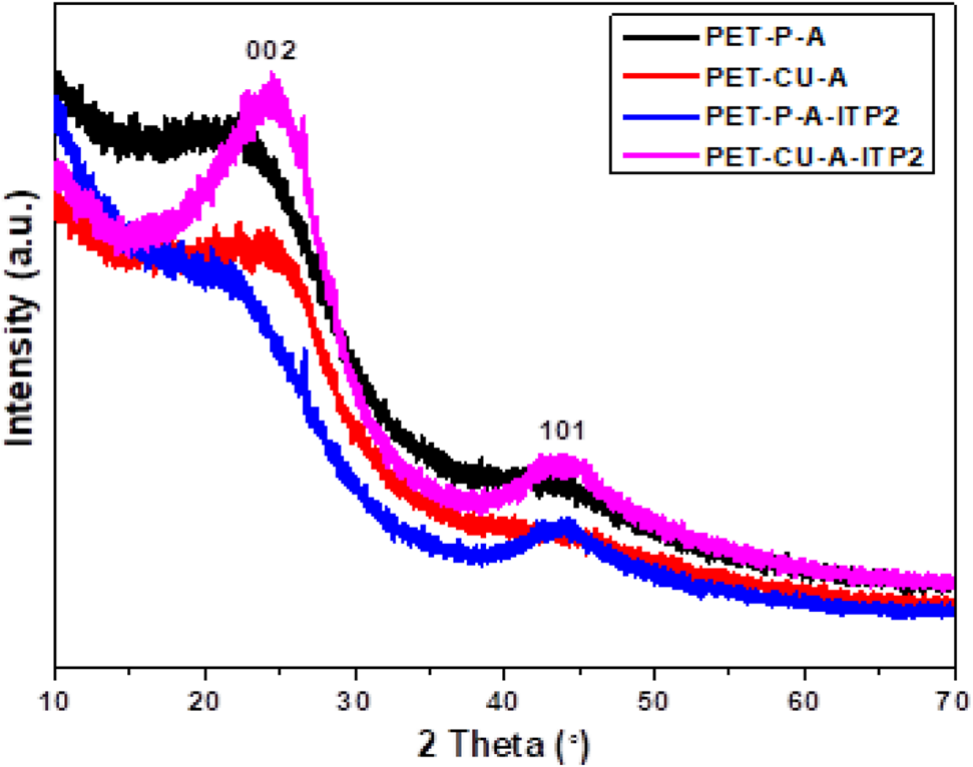
XRD patterns of activated carbons derived from PET using ITP method.

XRD was used to evaluate the crystallinity and graphitizableity of the carbon products and the XRD patterns are shown in Fig. 3. All patterns show two characteristic broad and weak peaks representing the 002 and 101 reflections, typical of amorphous, disordered pseudo-graphitic carbons.^25,36–38^ The 002 and 101 reflections can be attributed to the absence of ordered layered crystalline structure observed for graphite.^36,39^ The PET-CU-A-ITP2 sample shows the narrowest and most intense peaks of all samples indicating an enhanced stacking structure and higher degree of graphitization. This could be due to the presence of CU-DES which decomposes the PET at a much lower temperature (as seen in the TGA results) leading to the gradual growth of a 2D crystalline structure along the a and *c* axes.^37^ It should be noted that the PET-CU-A-ITP2 and PET-P-A-ITP2 samples presented the highest degree of carbonization as shown in the elemental analysis. PET-P-A-ITP2 gave the lowest and broadest 002 reflection which is accounting for the poorest layer-to-layer orientation in all the samples.^40^ In addition, the 002 reflections gradually shift from PET-P-A-ITP2 at around 21^°^ to higher angles (25^°^) for PET-CU-A-ITP2 which indicates the decrease of the interlayer spacing as a result of the increase in number of stacked carbon layers and a well-ordered carbon.^24,37^ The interlayer spacing, *d*_(002)_ was estimated using the Bragg equation (eqn (1)) and the values are shown in Table 2. All the results show that the interlayer spacing of the samples is higher than that of well-crystalline graphite (3.35 Å). It can be seen that PET-CU-A-ITP2 shows the lowest *d*_(002)_ value of 3.65 Å while PET-P-A-ITP2 gives the highest value of 4.14 Å. Other reports have shown a decrease in the value of *d*_(002)_ with increase in graphitization temperature with the more graphitized carbon displaying the smallest *d*_(002)_ values. These results show that the PET-CU-A-ITP2 sample has the highest degree of graphitization which can be linked to the use of CU-DES in its synthesis. This is also corroborated by the higher intensities of the (002) and (101) reflections revealing the enhanced stacking structure of the PET-CU-A-ITP2 carbons. The PET-CU-A sample shows the least pronounced 101 reflection indicative of most amorphous carbon compared to the other carbonaceous materials.^24^ The high level of graphitization might increase the electrochemical performance because of the enhanced electronic conductivity. The crystallite size or average width of the graphitic domain (*L*_a_) obtained from the 100 reflection and the stacking height or thickness of the graphitic domain (*L*_c_) obtained from the 002 reflection are both calculated using the Scherrer equation (eqn (2) and (3)).^37^ Surprisingly, the PET-CU-A-ITP2 carbons present the smallest *L*_a_ and *L*_c_ values of 1.70 nm and 0.61 nm, respectively of all the samples. This can be explained by the higher degree of graphitization (*i.e.* increased decomposition and condensation of organic groups in the PET precursor) in the PET-CU-A-ITP2 sample resulting in the smallest amounts of residues as shown in the elemental analysis (see Table 1). Furthermore, all XRD reflections display a raising background at low angle which can be due to the presence of large amounts of fine structure microporosity overlaying the 002 reflection.^36,38^1
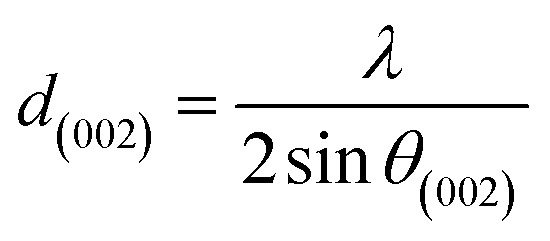
2
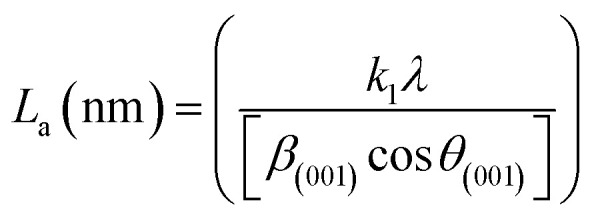
3
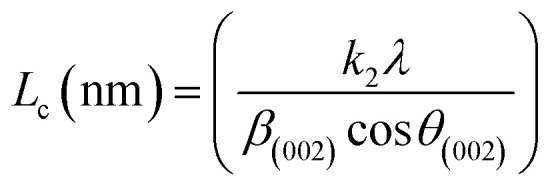


**Table 2:** Micro-structural properties obtained from XRD analysis

Sample	*d*_002_ a (Å)	*L*_a_ b (nm)	*L*_c_ c (nm)
PET-P-A	3.99	2.7	0.7
PET-CU-A	3.99	2.8	0.7
PET-P-A-ITP2	4.14	1.8	1.3
PET-CU-A-ITP2	3.65	1.7	0.6

a *d*_002_ is the average graphite interlayer spacing calculated from the 002 peak centers.

b *L*_a_ is the crystallite size of the graphitic domain obtained from 100 reflection.

c *L*_c_ is the stacking height obtained from 002 reflection.

### Raman spectroscopy

**Fig. 4:**
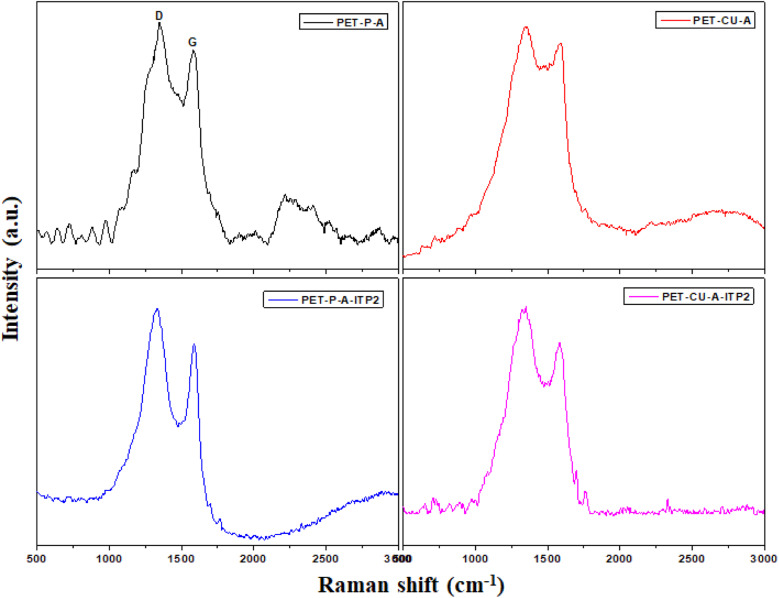
Raman spectra of activated carbons derived from waste PET.

Raman spectroscopy is a very important technique to measure the extent of disorder or local changes in the structure of various carbon materials.^41^ The Raman spectra of the obtained carbonaceous materials are given in Fig. 4. They all display the D (∼1347 cm^−1^) and G (∼1590 cm^−1^) bands characteristic of disordered and graphitic carbons. The D band is due to disorder or defects in the carbon structure while the G band is correlated to the stretching of the carbon atoms in the graphitic layer. The results of the intensity ratio *I*_D_/*I*_G_ are shown in Table 3. Surprisingly, the PET-CU-A-ITP2 sample shows the highest *I*_D_/*I*_G_ value of 1.21 which can indicate lowest level of graphitization degree or highest level of disordered/defect carbon structure.^25,37,42^ Clearly, the XRD results have shown that the PET-CU-A-ITP2 sample displayed the highest level of graphitization/ordering of all the samples. The elemental analysis showed that nitrogen is doped both into PET-CU-A and PET-CU-A-ITP2 materials which can also introduce defects in these materials. Furthermore, it is interesting that PET-CU-A-ITP2 material which is the closest to a turbostratic-type carbon has the highest *I*_D_/*I*_G_ value while PET-CU-A which is the closest to amorphous carbon has the lowest *I*_D_/*I*_G_ value (1.07). This implies that the *I*_D_/*I*_G_ value might also be a measure of the numeric density of interatomic distance which is sensitive to the size and microstructure of a crystallite.^43^ Nevertheless, all the samples including PET-CU-A-ITP2 are still highly defective graphitic materials. As observed in the XRD analysis, the crystallite size or average width of the graphitic domain (*L*_a_) can be determined from the Raman spectroscopy using eqn (4).^44^ The *L*_a_ results can be found in Table 3. Particularly, PET-CU-A-ITP2 gives the smallest crystallite size (3.64 nm) of all samples, in accordance with the XRD results. Both XRD and Raman spectroscopy analysis show that the PET-CU-A-ITP2 has the smallest crystallite size. It can be seen that the crystallite size values obtained from Raman spectroscopy are higher than those from XRD analysis as also reported elsewhere.^36,45^ The analysis of *L*_a_ value from Raman spectroscopy is based on propagation of phonons which is quite different from XRD technique that is based on diffraction phenomenon.^45^ For example, when a carbon layer is bent, there will be a reduced detection of the coherent domains by XRD whereas the phonon propagation is less inhibited. As a result, the *L*_a_ value obtained from Raman spectroscopy will be larger than that calculated from XRD.^45^4
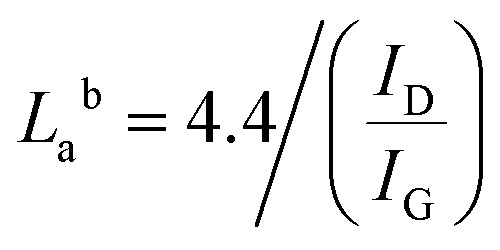


**Table 3:** Micro-structural properties obtained from Raman spectroscopy analysis

Sample	PET-P-A	PET-CU-A	PET-P-A-ITP2	PET-CU-A-ITP2
*I*_D_/*I*_G_^a^	1.14	1.07	1.18	1.21
*L*_a_ b (nm)	3.86	4.11	3.73	3.64

a I_D_ and *I*_G_ are the integrated intensities of the Raman D and G bands.

b L_a_ is the crystallite size obtained from Raman Spectroscopy.

### Pair distribution function analysis (PDF)

**Fig. 5:**
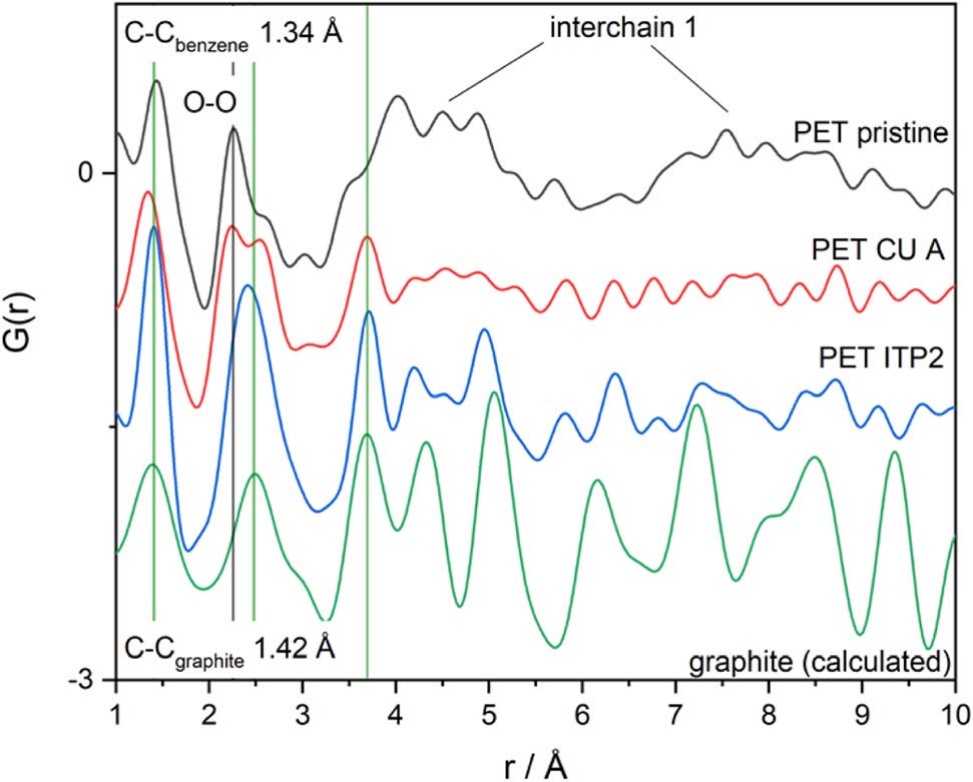
Pair distribution functions of PET-P, PET-CU-A, and PET-CU-A-ITP2 carbon materials compared with the calculated graphite structure.

The local atomic structures of PET-P, PET-CU-A, and PET-CU-A-ITP2 which were obtained from XRD patterns and are further analysed by PDF and compared with the calculated graphite structure as presented in Fig. 5. This figure shows the evolution of the graphitic structure from PET-P to PET-CU-A-ITP2 in terms of their local atomic structures and peak alignment with the calculated graphite structure. The PET-CU-A-ITP2 carbon shows three intense peaks associated with the real space distance of 1.40, 2.42 and 3.69 Å for the 1^st^, 2^nd^, and 3^rd^ peaks which aligns very well with those of the calculated graphite peaks seen at 1.42, 2.45, and 3.68 Å, respectively. It has been reported that the 1^st^ and 2^nd^ peaks correspond to the intralayer graphite distances while the 3^rd^ peak is associated with the interlayer graphite distances.^46^ Also, PET-CU-A-ITP2 shows virtually similar peaks to the calculated graphite peaks in the range above 4 Å; but PET-CU-A only displays a broad sinusoidal oscillation with no distinct peaks in this region indicating a higher degree of disorder in this material. In addition, the lower intensities of PET-CU-A compared with the PET-CU-A-ITP2 and the calculated graphite peaks imply that it has a higher amount of defects/disorder resulting from the high number of non-hexagonal carbon rings in its structure.^47^ The PDF analysis shows that the PET-CU-A-ITP2 carbon material displays a better graphite-like layered structure and less disorder compared to the other samples as also shown in the Raman spectroscopy results.

### Adsorption analysis of the carbonaceous materials: Brunauer Emmett Teller (BET) method

The surface area of the carbonaceous materials was determined using nitrogen adsorption. The adsorption isotherm up to 0.30 relative pressure was used to determine the surface areas using the BET equation. The N_2_ isotherms of the samples are shown in Fig. S1† with similar trends. The adsorption amounts increase gradually starting from the low pressure with the micropores filled with N_2_ gas at high pressure. At the knee, more evident in the PET-P-A and PET-P-A-ITP2 isotherms, a monolayer begins to form. According to IUPAC classification, the adsorption isotherms resemble that of type I/IV behaviour.^36,42^ As shown in Table 4, the BET surface area of PET-P-A (328.5 m^2^ g^−1^) and PET-P-A-ITP2 (502.9 m^2^ g^−1^) are significantly higher than those of PET-CU-A (8.5 m^2^ g^−1^) and PET-CU-A-ITP2 (23.1 m^2^ g^−1^). It is clear that the presence of CU-DES must have clogged the micropores of the PET-CU-A and PET-CU-A-ITP2 samples. In order to simplify the synthesis process and due to the assumption that the pyrolysis heating will evaporate any remaining CU-DES or other contaminants, no post-synthesis washing of the samples was done. Most reports use solvents such as acetone, alcohol, acids, and water to wash the post-synthesised carbon products which complicates the process, results in the usage of toxic chemicals, and increases the cost of the materials.^25,34,35,48^ In addition, apart from post-synthesis washing, the results show that higher pyrolysis temperature may be required for the CU-DES treated samples since enhanced surface area is recorded for PET-CU-A-ITP2 compared to PET-CU-A.

**Table 4:** BET surface areas of the activated carbons derived from PET

Sample	PET-P-A	PET-CU-A	PET-P-A-ITP2	PET-CU-A-ITP2
BET surface area (m^2^ g^−1^)	328.5	8.5	502.9	23.1

### Morphological Analysis of activated carbons derived from PET

**Fig. 6:**
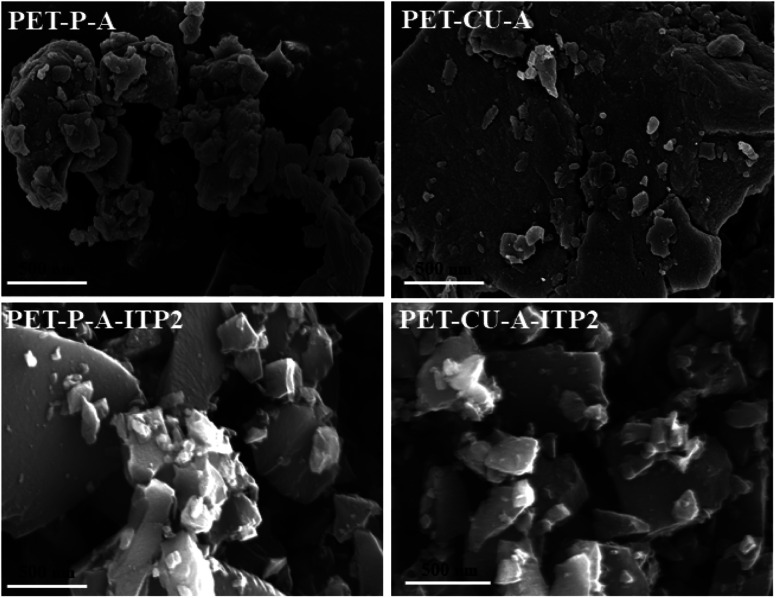
SEM micrographs of activated carbons derived from PET.

SEM was undertaken to study the morphology of the obtained activated carbons and they are shown in Fig. 6. PET-CU-A shows a highly compact structure which confirms its smallest BET surface area compared to the other samples. PET-P-A consists of agglomerated particles with few smaller particles at the surface and reveals the presence of some pores. After pyrolysis, PET-P-A-ITP2 and PET-CU-A-ITP2 show obvious morphological changes with both samples displaying larger irregular non-agglomerated particles with few smaller particles at the surface on top of them. They also show some porosity.

**Fig. 7:**
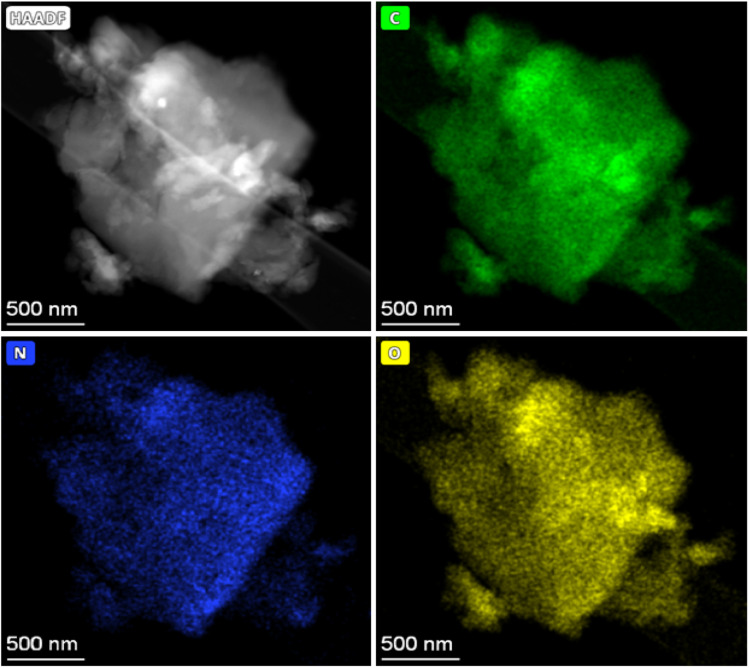
STEM image of PET-P-A carbons using HAADF detector and its corresponding EDX mapping showing the distribution of C, N, and O elements in the materials.

EDX analysis was used to show the elemental distribution and mapping in the carbonaceous materials. Fig. 7 shows the STEM-EDX images of PET-P-A carbons and its corresponding EDX mapping which shows the distribution of C, N, and O elements in the materials. The STEM image shows an agglomerated particle as also revealed by the SEM analysis. Fig. S2† gives the SEM image of activated carbon powders derived from PET and their corresponding EDX mapping showing the homogeneous distribution of C, N, and O elements in the material. The elemental composition of C, N, and O elements largely agrees with the elemental distribution from the CHN analysis. STEM-EDX was used for PET-P-A to determine the elemental mapping of the material which corroborates the SEM-EDX analysis.

## Electrochemical performance

### Cyclic voltammetry (CV)

**Fig. 8:**
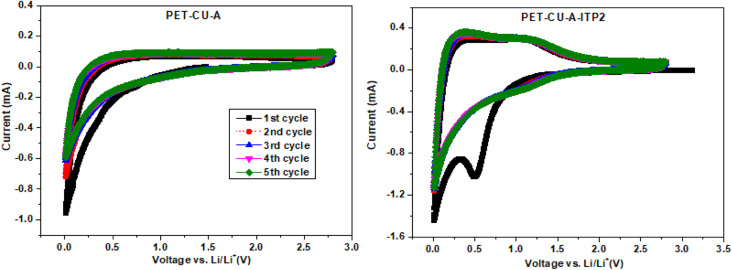
Cyclic voltammetry of the PET-CU-A and PET-CU-A-ITP2 carbon anodes measured at a scan rate of 0.1 mV s^−1^ between 0.01 - 2.8 V (*vs.* Li/Li^+^) for five cycles at 25 °C.

CV measurements were used to elucidate the electrochemical behaviour and to determine the mechanism of capacitive contributions of PET-CU-A and PET-CU-A-ITP2 carbon anodes in a LIB. Fig. 8 shows the CV curves which were performed at a scan rate of 0.1 mV s^−1^ between the cut-off voltages of 0.01 and 2.8 V (*vs.* Li/Li^+^) at 25 °C. Typically, in CV, the positive current describes lithium de-intercalation while the negative current gives the lithium intercalation into the anode. The CV curves shows that Li^+^ can reversibly (de-)intercalate into the carbon anode materials. The Li^+^ storage mechanism in carbon materials is based on its reversible intercalation in defective graphene layers, nanopore filling, and reaction with heteroatoms.^42,49^ The increase in current at lower voltages shows the beginning of electrolyte decomposition leading to solid electrolyte interphase (SEI) formation as shown in other reports.^42,50,51^ The CV curve of PET-CU-A shows no sharp peak in contrast to that of PET-CU-A-ITP2 indicating less electrochemical activity. The 1^st^ cycle of PET-CU-A-ITP2 shows a broad reduction peak at about 0.5 V which describes the decomposition of electrolyte on the anode surface during Li^+^ insertion forming the SEI film. However, this peak disappears in the subsequent cycles showing curves that are overlapped, indicating stable SEI film on the anode surface and the reversibility of the electrochemical process. It has been shown that such a stable SEI film can protect the electrolyte from further degradation during subsequent charge–discharge cycles.^52^ Furthermore, the one pair of weak reduction and oxidation peaks between 1.0–1.5 V (2^nd^–5^th^ cycle), not seen in the PET-CU-A anode, corresponds to Li^+^ (de−)intercalation from the defective graphene layers. The current flow that occurs in the voltage range of 0.1–1.0 V is due to the reaction of Li^+^ with N heteroatoms doped into the carbon materials by using CU-DES while the current flow observed close to 0.1 V is a result of nanopore filling.^51^ In addition, the higher peak height and area in the CV of PET-CU-A-ITP2 anode confirms the better electrochemical performance compared to PET-CU-A anode. The results agree with the XRD results which predicted enhanced electrochemical performance for the PET-CU-A-ITP2 anode since it presented the highest level of graphitization and the most enhanced stacking structure of all the carbon materials.

### Galvanostatic charge–discharge profiles

**Fig. 9:**
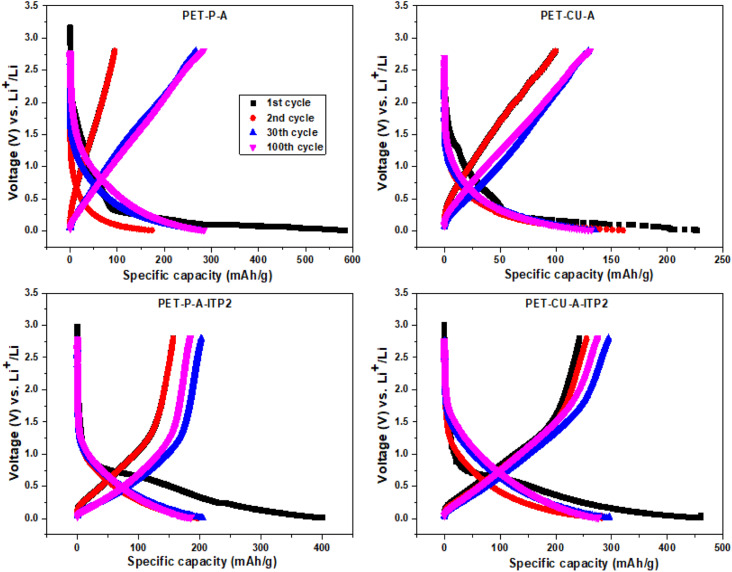
Galvanostatic charge–discharge profiles (1^st^, 2^nd^, 30^th^, and 100^th^ cycles) of the carbon anode materials derived from PET cycled at a current density of 100 mA g^−1^ between 0.01 - 2.8 V (*vs.* Li/Li^+^) at 25 °C.

The galvanostatic charge–discharge profiles of the obtained carbon materials used as anode in LIB and cycled at a current density of 100 mA g^−1^ between 0.01 - 2.8 V (*vs.* Li/Li^+^) at 25 °C are shown in Fig. 9. It is important to note that no initial activation was applied at a lower current density. The quantitative electrochemical performance data for the 1st, 2^nd^, 30^th^, and 100^th^ cycles are given in Table S1.† All the profiles follow the typical charge–discharge curves of pseudo-graphitic carbonaceous materials used as anodes in LIBs with an extended plateau in the 1^st^ discharge curves.^48,53^ The PET-P-A, PET-CU-A, PET-P-A-ITP2, and PET-CU-A-ITP2 anodes give 1^st^ cycle discharge capacities of about 584, 227, 401, and 461 mA h g^−1^ and 1^st^ cycle coulombic efficiency (CE) of about 16%, 24%, 39%, and 53%, respectively. This means that the PET-P-A anode shows the largest 1^st^ cycle capacity loss (∼489 mA h g^−1^) while the PET-CU-A-ITP2 anode gave the smallest capacity loss (∼218 mA h g^−1^). These large irreversible 1^st^ cycle capacity losses accompanied with low CE are typical of carbonaceous anode materials as a result of the decomposition of electrolytes and subsequent formation of SEI on the anode surfaces.^53^ Also, the disordering in the carbon structure has been implicated for this large 1^st^ cycle capacity loss since Li^+^ could be trapped in the voids of the carbon matrix.^54^ This is in agreement with the XRD analysis that showed that PET-P-A (along with PET-CU-A) displayed highly disordered carbon structure which could be accounting for their larger capacity loss and lower CE.

It can be seen that for all the anodes in the 2^nd^ cycle (Table S1†), there is a reduction in the discharge capacities and capacity losses with concomitant increase in the CE. These trends continued till after the 100^th^ cycle except that the discharge capacities of the anodes generally increase from the 2^nd^ cycle which could be due to enhanced structural stability of the anode materials after SEI formation in the 1^st^ cycle. For example, the PET-P-A anode recorded a discharge capacity and CE of ∼174 mA h g^−1^ and ∼60% in the 2^nd^ cycle, while after the 100^th^ cycle it showed ∼288 mA h g^−1^ and ∼90%, respectively. Similarly, the PET-CU-A-ITP2 anode recorded a discharge capacity and CE of ∼283 mA h g^−1^ and ∼90% in the 2^nd^ cycle, while after the 100^th^ cycle it showed ∼276 mA h g^−1^ and ∼100%, respectively. Comparatively, the ITP carbon anode materials (PET-P-A-ITP2 and PET-CU-A-ITP2) gave better electrochemical performance than the annealed analogues (*i.e.* PET-P-A and PET-CU-A) which can be traced to their higher graphitization and ordering.

The galvanostatic charge–discharge profiles of the carbon anode materials were also cycled at a current density of 50 mA g^−1^ between 0.01–2.8 V (*vs.* Li/Li^+^) at 25 °C as shown in Fig. S3.† The quantitative electrochemical performance data for the 1^st^ and 2^nd^ cycles are given in Table S2.† It is well known that the cycling current density can be used to control the kinetics of the electrochemical process. Usually, more Li^+^ can be (de)intercalated into the electrode materials at lower charge current density, thereby increasing the capacity and CE.^35^Table 4 6 shows that the PET-P-A, PET-CU-A, PET-P-A-ITP2 and PET-CU-A-ITP2 anodes cycled with a current density of 50 mA g^−1^ gave 1^st^ cycle discharge capacities of about 1191, 769, 636, and 650 mA h g^−1^ and CE of about 20%, 47%, 46%, and 58%, respectively; which were all significantly higher than the values obtained when these anodes were cycled at 100 mA g^−1^. A similar trend can also be seen with the values obtained in the 2^nd^ cycle. Like the ones cycled at 100 mA g^−1^, there was a reduction in the discharge capacities and capacity losses with concomitant increase in the CE for all the anodes. Overall, the PET-CU-A-ITP2 anode gave the best electrochemical performances at both current densities.

### Cycling performance

**Fig. 10:**
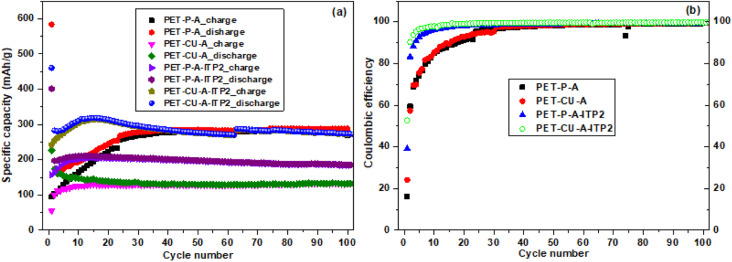
Electrochemical performance of the carbon anode materials derived from PET cycled at a current density of 100 mA g^−1^ between 0.01–2. 8 V (*vs.* Li/Li^+^) at 25 °C (a) Charge/discharge as a function of cycle number (b) coulombic efficiency as a function of cycle number.

The cycling performance and coulombic efficiency of the carbon anode materials derived from PET cycled at a current density of 100 mA g^−1^ between 0.01 - 2.8 V (*vs.* Li/Li^+^) at 25 °C are shown in Fig. 10. All the carbon anodes show stable cycling with only weak capacity degradation as shown in Fig. 10a. A reduction of discharge capacity is seen after the 1^st^ cycle which increased generally after the 2^nd^ cycle. Particularly, the capacity retention (from 2^nd^ to 100 cycles) for the PET-P-A, PET-CU-A, PET-P-A-ITP2, and PET-CU-A-ITP2 anodes is 166%, 76%, 94% and 98%, respectively. The 1^st^ cycle CE increased from ∼16% in PET-P-A anode to 53% in PET-CU-A-ITP2 anode. The PET-CU-A and PET-P-A-ITP2 anodes show an average stable charge–discharge capacity of ∼135 and ∼200 mA h/g over 100 cycles. The PET-P-A and PET-CU-A-ITP2 anodes presented higher average stable charge–discharge capacities of ∼295 mA h g^−1^ for both anodes. Fig. 10b shows that the CE increased steadily for all the anode materials, but only the PET-CU-A-ITP2 anode reached ∼90% in the 2^nd^ cycle and ∼100% from 20^th^–100^th^ cycle indicating better stabilisation of the SEI.^55^ The PET-P-A and PET-CU-A anodes had very poor CE mostly in the first 30 cycles as also seen in the non-overlap of the charge–discharge curve in Fig. 10a.

### Rate performance

**Fig. 11:**
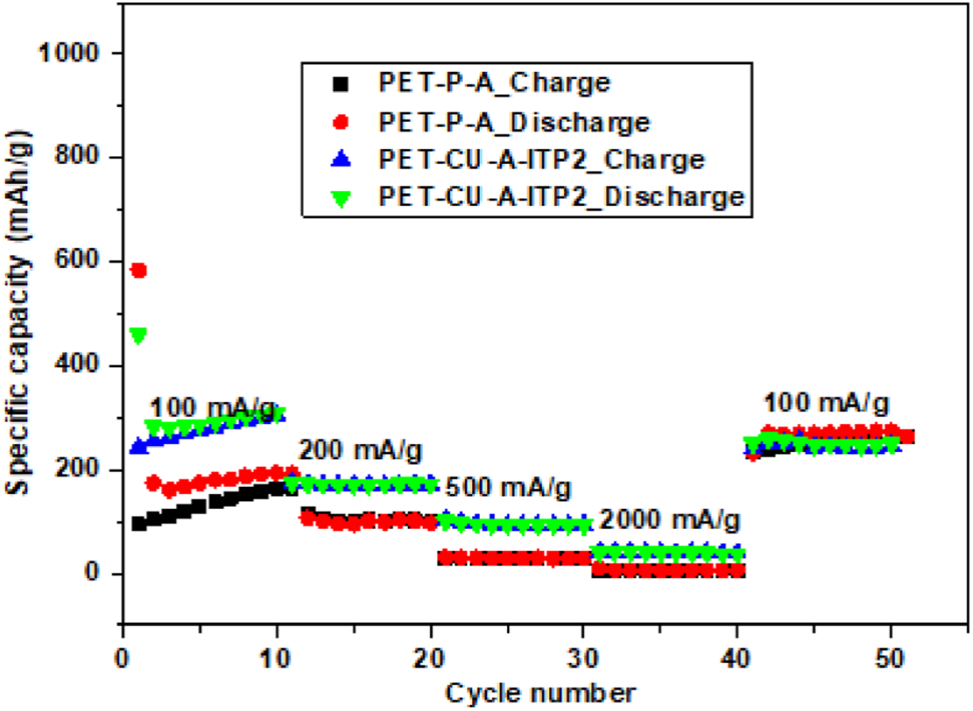
Rate performance tests of PET-P-A and PET-CU-A-ITP2 anodes cycled at varying current densities (100–2000 mA g^−1^) between 0.01–2.8 V (*vs.* Li/Li^+^) at 25 °C.

The rate performances of PET-P-A and PET-CU-A-ITP2 anodes were determined by performing galvanostatic charge–discharge tests for 10 cycles at different current density ranging from 100–2000 mA g^−1^ and it is shown in Fig. 11. The galvanostatic charge–discharge profiles (10^th^ cycle) of these two anodes are given in Fig. S4.† These two anode materials were selected since they presented the best combination of specific capacity and capacity retention. Particularly, the two anode materials allowed for the comparison between the influence of the method of synthesis (annealing and pyrolysis) and the use/non-use of CU-DES on both their electrochemical performances. Using the 2^nd^ cycles, the PET-CU-A-ITP2 anode delivered specific capacities of 283, 172, 97, and 42 mA h g- 2 while the PET-P-A anode delivered specific capacities of 174, 106, 30, and 6 mA h g- 2 at current densities of 100, 200, 500, and 2000 mA g^−1^, respectively. This result clearly shows that the rate performance of the PET-CU-A-ITP2 anode is better than that of the PET-P-A anode at all current densities tested. However, both anodes displayed good reversibility and virtually same specific capacity when they were cycled at 100 mA g^−1^ immediately after cycling at 2000 mA g^−1^.

## Discussion

The electrochemical test results show clearly that PET-CU-A presented the poorest performance of all materials. This can be connected to its structure and composition. The XRD results show that PET-CU-A had the highest disorder as seen in the 101 reflection which was the least pronounced of all the samples.^24^ Also, the poorest electrochemical performance of this material could be traced to its highly compact structure and its smallest BET surface area compared to the other carbon materials. However, the high initial capacity of PET-CU-A carbon could be caused by the highest amount of N doped into its structure. It is well known that N doping can induce defects, increase available active sites, and can improve the electronic and chemical characteristics, resulting in the enhancement of electrochemical reactivity in LIBs.^42,50,56^ Generally, the PET-P-A anode displayed better electrochemical performance compared to PET-P-A-ITP2 anode; even though it had lower surface area, lesser graphitization/ordering and undesired poor CE which was the poorest of all the carbon anodes. Poor CE is due to the irreversible trapping of Li^+^ in the highly disordered carbon and amorphous regions. The generally poorer performance of the PET-P-A-ITP2 anode can only be explained from its poorest layer-to-layer orientation in all the samples which can influence Li^+^ kinetics in the anode.

The elemental analysis results show that PET-CU-A-ITP2 carbon (along with PET-P-A-ITP2) shows the highest amount of carbon indicating the highest level of carbonization. This was also confirmed by the XRD results which showed that PET-CU-A-ITP2 has enhanced stacking structure and higher degree of graphitization compared to other samples. This high level of graphitization and the best layer-to-layer orientation in PET-CU-A-ITP2 carbons conferred enhanced electronic conductivity which increased the electrochemical performance compared to the other carbon materials. The specific capacity in these carbons can be due to reactions with the heteroatom, reversible adsorption on defective sites, intercalation in the graphitic sheets, and nanopore filling.^42,50,56^ The different charge storage mechanisms and the contributions to the specific capacity of the carbon anodes can be obtained from the plateau and slope of the galvanostatic discharge profiles. There are conflicting reports in literature on the assignment of the particular charge storage mechanisms to the plateau and slope regions of the galvanostatic discharge profiles of pseudo-graphitic carbon materials. While some researchers showed that Li^+^ ion intercalation in the graphitic sheets was dominant in the sloping region (above 0.1 V),^57,58^ others demonstrated that this same storage mechanism accounted for the plateau region (below 0.1 V).^42,50^ Furthermore, the plateau region (below 0.1 V) has been described based on nanopore filling^56^ while others showed that no nanopore filling was observed even at 0.001 V.^42^ Yet other researchers have shown that reversible binding on defects, surfaces and edges accounts for the sloping region.^42,47,50^ However, it is clear that the total capacity of these pseudo-graphitic carbon materials is a sum of different charge storage mechanisms as shown above.

**Fig. 12:**
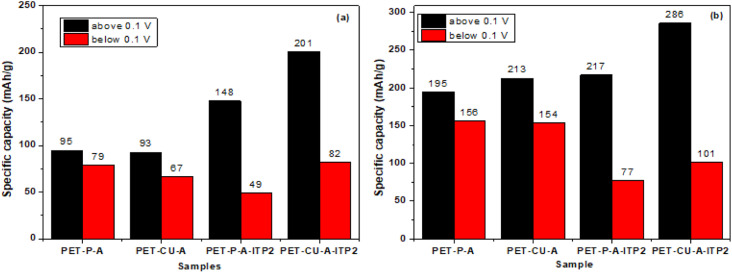
Capacity contributions from above 0.1 V (sloping region) and below 0.1 V (plateau region) of the 2^nd^ cycle discharge profiles at both 100 mA g^−1^ (a) and 50 mA g^−1^ (b).

Fig. 12 gives the capacity contributions from above 0.1 V (sloping region) and below 0.1 V (plateau region) of the 2^nd^ cycle discharge profiles at both 50 and 100 mA g^−1^. The results show that the sloping region contributes higher capacity compared to the plateau region in all the carbon anodes at both current densities. The PET-CU-A-ITP2 and PET-P-A-ITP2 carbons show higher capacity values in the sloping region at both current densities. This might be due to the higher levels of graphitization/ordering in these materials as seen from their XRD analysis which favours intercalation in the graphitic sheets. In contrast, the PET-P-A and PET-CU-A carbons generally present higher capacity values in the plateau region at both current densities. Since these carbons displayed lower graphitization/ordering compared to the PET-CU-A-ITP2 and PET-P-A-ITP2 carbons, these defects must be the dominant charge storage centers in this region. The N doping on the PET-CU-A-ITP2 carbon which can induce defects or increase available active sites may be responsible for the capacity values in the plateau region comparable to PET-P-A and PET-CU-A carbons. These results also show that the surface area had no clear contributions to the total capacity since PET-P-A-ITP2 carbon with the highest surface area (502.9 m^2^ g^−1^) presents a smaller capacity than PET-CU-A-ITP2 carbon with a surface area of 23.1 m^2^ g^−1^. Excellent electrochemical performance has been reported for highly volumetrically densified banana peels-derived carbons with low surface areas which aided excellent electrode packing characteristics, a high volumetric capacity, and low levels of SEI formation.^42^ Overall, the PET-CU-A-ITP2 carbons display the highest total capacities in both the sloping and plateau regions at both current densities. The superior electrochemical performance of the PET-CU-A-ITP2 carbon anode could be due to its low surface area which can result in better electrode packing, higher capacity, less SEI formation and better CE. Even though the PET-CU-A-ITP2 carbon anode recorded small initial CE (53%), this value is comparable with the one obtained from other carbons such as graphene anode^59^ and *N*-doped graphitic carbon anode.^60^ These results show that dense carbons can be exploited as high-performance anode in LIBs and can further be improved by increasing the pyrolysis temperature. The use of CU-DES in a facile synthesis to prepare pseudo-graphitic carbons from abundant waste PET bottles could facilitate its commercial application since it is cheap and environmentally friendly. Also, this type of waste-to-energy approach will not only help in the sustainable management of these wastes but could also offer cheaper and greener LIBs to tackle the energy poverty presently faced by most African countries. The electrochemical performance of PET-CU-A-ITP2 carbon in LIB has been compared with a range of various types of carbon anodes in LIB and is shown in Table S3.†

## Conclusion

In summary, pseudographitic activated carbons (AC) were prepared from waste PET bottles using ionothermal pyrolysis. The use of CU-DES as the solvent medium and nitrogen source enhanced the simplicity, reduced toxicity and resulted in low-cost ACs which could facilitate the large-scale production of this important functional material. After different optimization steps, the pre-stabilization of PET in air was found to be critical for its pyrolysis to obtain AC. Also, it was found that on extending the pre-stabilization step for a longer duration, AC material (PET-P-A and PET-CU-A) were produced which gave good electrochemical performance comparable to the ones produced from the ITP method. This method of producing AC from PET using low temperature annealing in the presence of air can even further simplify the synthesis process and the cost of AC. Furthermore, the use of CU-DES created low-surface area ACs and higher levels of graphitization/ordering in the ACs. The obtained ACs were applied as anodes in LIBs. The PET-CU-A-ITP2 gave the best electrochemical performance that could be upscaled to meet the requirements for commercial application. The results show that the electrochemical parameters such as initial coulombic efficiency, specific capacity and cycling performance are controlled by the microstructural properties of the AC materials which in turn are dependent on the method of synthesis and key parameters like the use of CU-DES. Comparing the electrochemical performance of PET-CU-A-ITP2 anode with the state-of-the-art lithium-ion batteries (LIBs) shows that it is promising and can be improved to meet large-scale LIBs application.

## Data availability

All data of this study are available from the corresponding author upon request.

## Author contributions

Ehi-Eromosele C.O. and Indris S. conceived, planned and supervised the experiments. Ehrenberg H. was the Chief Host and provided the research resources. Onwucha C.N. performed the synthesis and the electrochemical tests while Ajayi S.A. analysed the electrochemical data. Melinte G. and Hansen A.-L. performed the STEM and PDF experiments and analysis, respectively. Ehi-Eromosele C.O. wrote the first draft while all authors reviewed the manuscript.

## Conflicts of interest

There are no conflicts of interest to declare.

## Supplementary Material

RA-012-D2RA06786B-s001
