# Supplementary material for: Ionothermal synthesis of activated carbon from waste PET bottles as anode materials for lithium-ion batteries
Source: RSC Adv. 2022 Dec 2;12(53):34670–84. doi: 10.1039/d2ra06786b (PMC9717347; doi:10.1039/d2ra06786b)
Supplement: RA-012-D2RA06786B-s001 [file RA-012-D2RA06786B-s001.pdf]

Electronic Supplementary Material (ESI) for RSC Advances.
This journal is © The Royal Society of Chemistry 2022

Ionothermal Synthesis of Activated Carbon from Waste PET Bottles as Anode Materials for Lithium-Ion Batteries

Cyril O. Ehi-Eromosele[1,2,*], Chizoom N. Onwucha[1,2], Samuel O. Ajayi[1,2], Georgian Melinte[4], Anna-Lena Hansen[1], Sylvio Indris[1,3], Helmut Ehrenberg[1,3]

[1]Institute for Applied Materials (IAM), Karlsruhe Institute of Technology (KIT), 76344 Eggenstein-Leopoldshafen, Germany.
[2]Department of Chemistry, Covenant University, PMB 1023, Ota, Nigeria
[3]Helmholtz Institute Ulm (HIU) Electrochemical Energy Storage, Helmholtzstrasse 11, 89081 Ulm, Germany.
[4]Institute of Nanotechnology (INT), Karlsruhe Institute of Technology (KIT), Hermann-von-Helmholtz-Platz 1, 76344 Eggenstein-Leopoldshafen, Germany.

Corresponding Author
[*]Email: cyril.ehi-eromosele@covenantuniversity.edu.ng

SUPPORTING INFORMATION

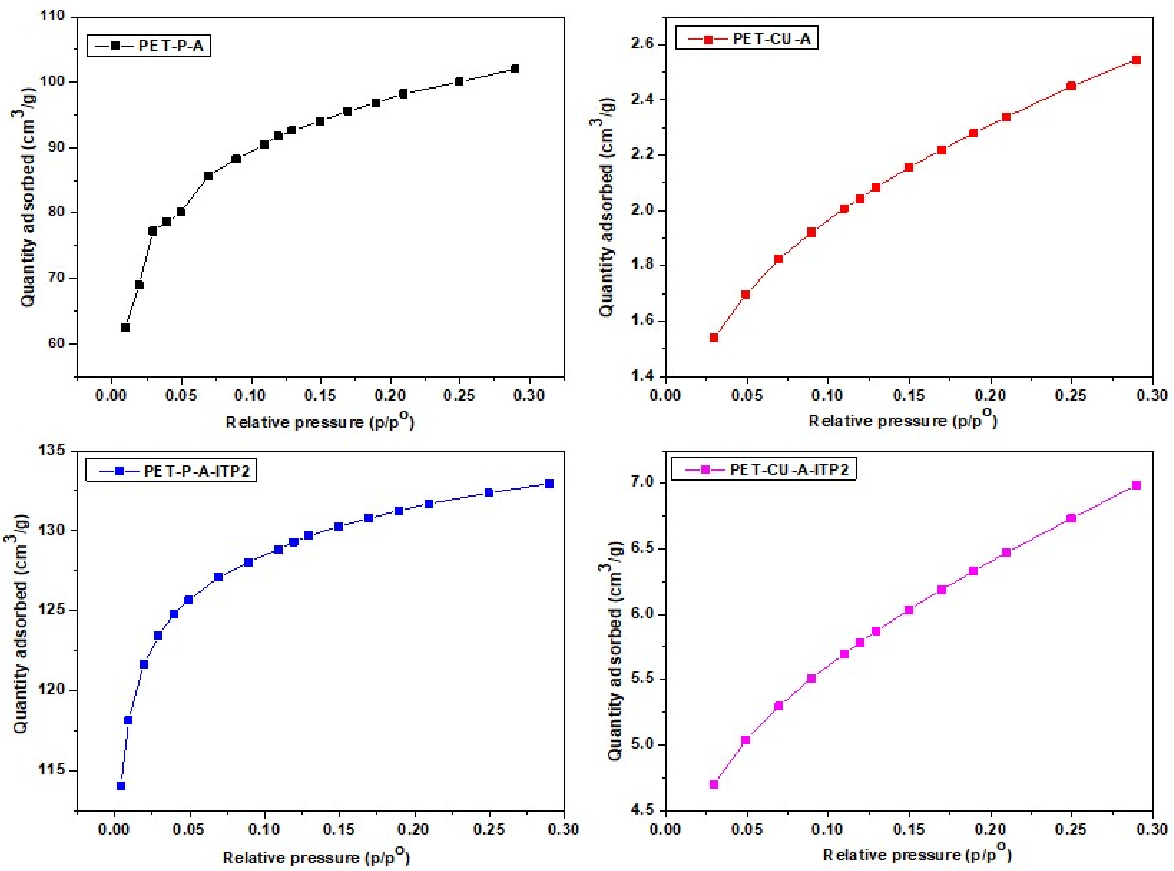

Figure S1. Nitrogen adsorption isotherms of activated carbons derived from PET

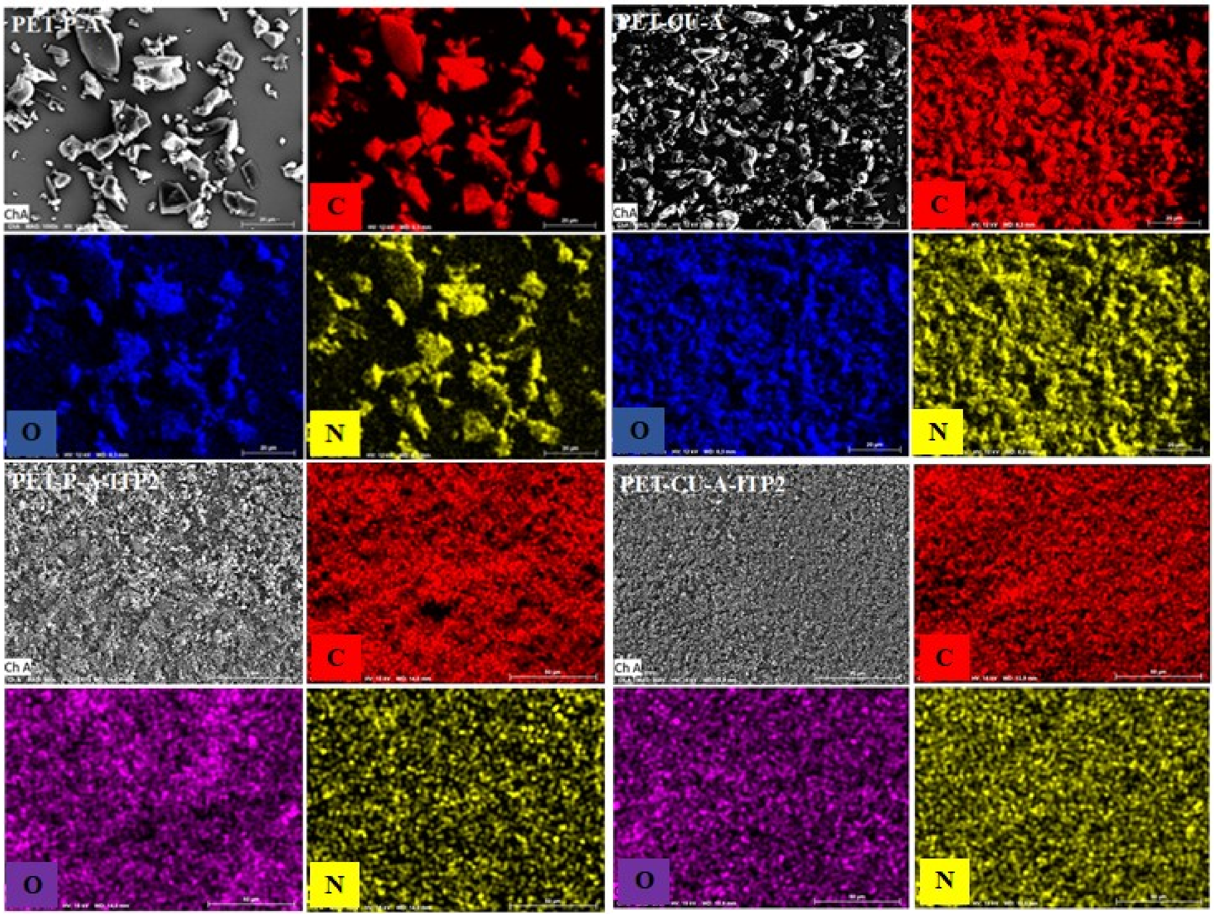

Figure S2. SEM images of activated carbons derived from PET and their corresponding EDX mapping showing the distribution of C, N, and O elements in the materials.

Table S1. Electrochemical performance data of the charge-discharge curves for the 1st, 2nd, 30th, and 100th cycles of the carbon anode materials derived from PET cycled at a current density of 100 mA/g between 0.01 - 2.8 V (vs. Li/Li^+) at 25°C.

	**Samples**	**PET-P-A**	**PET-CU-A**	**PET-P-A-ITP2**	**PET-CU-A-ITP2**
1st cycle	Charge capacity (mAh/g)	94.44	54.57	156.88	242.81
	Discharge capacity (mAh/g)	583.65	226.67	401.18	460.65
	Capacity Loss (mAh/g)	489.21	172.10	244.30	217.84
	Coulombic efficiency (%)	16.18	24.08	39.11	52.71
2nd cycle	Charge capacity (mAh/g)	103.36	99.62	164.23	255.23
	Discharge capacity (mAh/g)	173.77	160.26	197.31	282.65
	Capacity Loss (mAh/g)	70.41	60.64	33.08	27.42
	Coulombic efficiency (%)	59.48	62.16	83.24	90.30
30th cycle	Charge capacity (mAh/g)	268.61	129.20	202.20	294.32
	Discharge capacity (mAh/g)	278.51	135.42	205.07	295.99
	Capacity Loss (mAh/g)	9.90	6.22	2.87	1.67
	Coulombic efficiency (%)	96.45	95.41	98.60	99.44
100th cycle	Charge capacity (mAh/g)	268.37	131.46	184.19	275.40
	Discharge capacity (mAh/g)	288.16	131.74	185.48	276.11
	Capacity Loss (mAh/g)	19.79	0.28	1.29	0.71
	Coulombic efficiency (%)	93.13	99.79	99.31	99.74

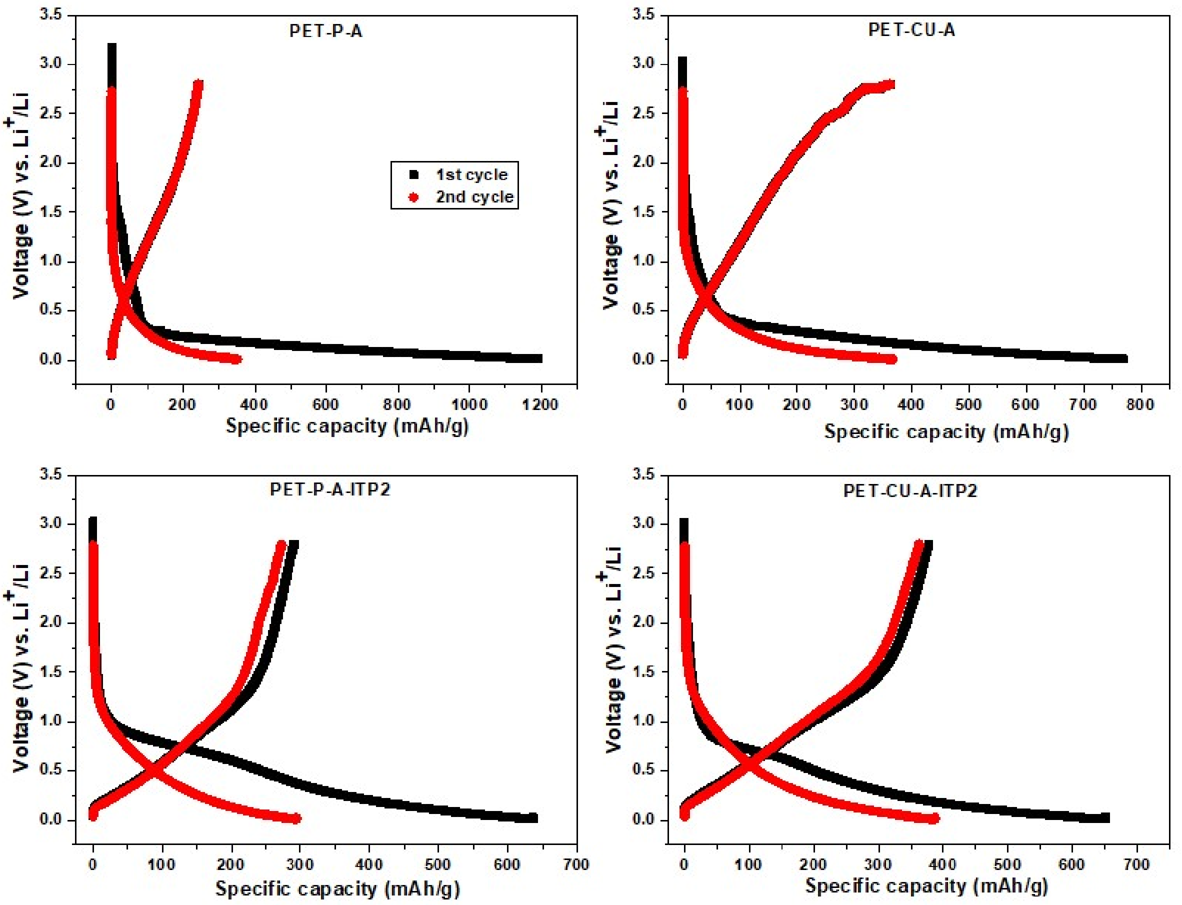

Figure S3: Galvanostatic charge-discharge profiles (1st and 2nd cycles) of the carbon anode materials derived from PET cycled at a current density of 50 mA/g between 0.01 - 2.8 V (vs. Li/Li^{+}) at 25°C.

Table S2. Electrochemical performance data of the charge-discharge curves for the 1st and 2nd cycles of the carbon anode materials derived from PET cycled at a current density of 50 mA/g between 0.01 - 2.8 V (vs. Li/Li^{+}) at 25°C.

	**Samples**	**PET-P-A**	**PET-CU-A**	**PET-P-A-ITP2**	**PET-CU-A-ITP2**
1st cycle	Charge capacity (mAh/g)	242.60	361.50	290.57	377.23
	Discharge capacity (mAh/g)	1190.76	768.48	636.06	650.42
	Capacity Loss (mAh/g)	948.16	406.98	345.49	273.19
	Coulombic efficiency (%)	20.37	47.04	45.68	58.00
2nd cycle	Charge capacity (mAh/g)	242.60	361.50	272.67	362.41
	Discharge capacity (mAh/g)	351.40	367.26	294.12	386.71
	Capacity Loss (mAh/g)	108.80	5.76	21.45	24.30
	Coulombic efficiency (%)	69.04	98.43	92.71	93.72

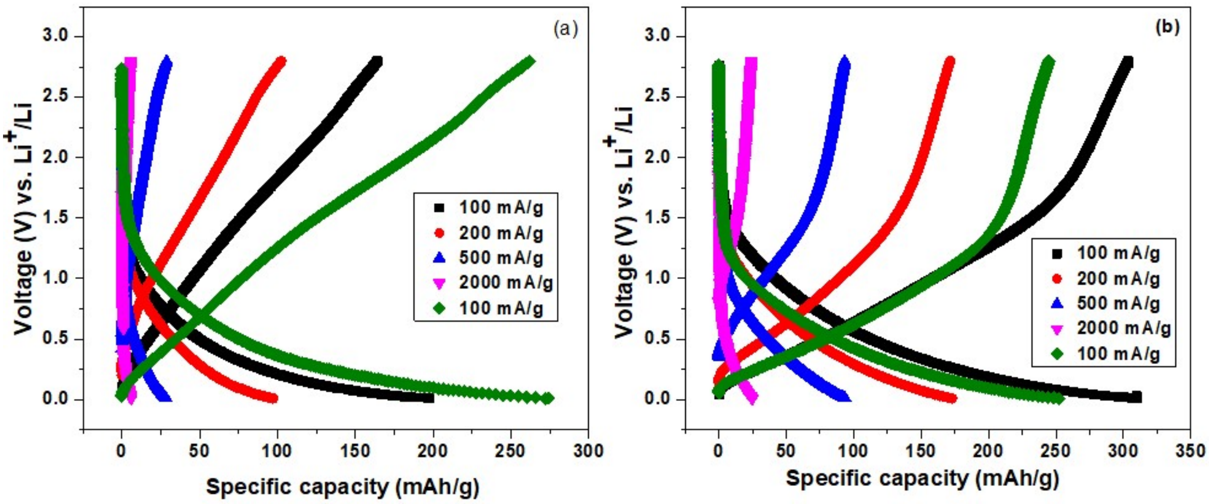

Figure S4: Rate performance test - galvanostatic charge-discharge profiles (10th cycle) of PET-P-A and PET-CU-A-ITP2 anodes cycled at varying current densities (100 – 2000 mA/g) between 0.01 - 2.8 V (vs. Li/Li^+) at 25 ^oC.

Table S3. Comparison of PET-CU-A-ITP2 carbon with the state-of-the-art LIBs

Material	Initial Coulombic Efficiency (%)	Discharge capacity (2nd cycle) mAh/g	References
PET-CU-A-ITP2	58	387 at 50 mA/g	This work
Carbon nanoparticles	55	742 at 100 mA/g	[1]
Porous carbon nanofibres	66	491 at 50 mA/g	[2]
Carbon nanofibres	NA	483 at 50 mA/g	[3]
Banana peel derived carbon	69	826 at 50 mA/g	[4]
Graphene	38	580 at 25 mA/g	[5]
Nitrogen doped graphitic carbons	49	840 at 50 mA/g	[6]
Carbon aerogels	63	310 at C/10	[7]

References

1 R. R. Gaddam, D. Yang, R. Narayan, K. V. S. N. Raju, N. A. Kumar, X. S. Zhao, *Nano Energy*, 2016, **26**, 346-352.

2 J. Liwen, Z. Xiangwu, *Nanotechnology*, 2009, **20**, 155705.

3 L. Ji, X. Zhang, *Electrochem. Commun.*, 2009, **11**, 684– 687.

4 E. M. Lotfabad, J. Ding, K. Cui, A. Kohandehghan, W. P. Kalisvaart, M. Hazelton, D. Mitlin, *ACS Nano*, 2014, ***8*(7)**, 7115-7129.

5 A. Kumar, A. L. M. Reddy, A. Mukherjee, M. Dubey, X. Zhan, N. Singh, L. Ci, W. E. Billups, J. Nagurny, G. Mital, P. M. Ajayan, *ACS Nano*, 2011, **5**, 4345-4349.

6 S-X. Wang, S. Chen, Q. Wei, X. Zhang, S. Y. Wong, S. Sun, X. Li, *Chemistry of Materials*, 2015, **27**, 336–342.

7 A. S. Alex, M. S. Ananda Lekshimi, V. Sekkar, B. John, C. Gouri, S. A. Ilangovan, Polymers *for Advanced Technologies,* 2017, https://doi.org/10.1002/pat.4085.
